# Sex and Relationships Education for Individuals with Cystic Fibrosis: A Service-Based Approach

**DOI:** 10.1007/s11195-018-9535-y

**Published:** 2018-10-22

**Authors:** Emily Norris, Samantha Phillips, Catherine Butler, Kirsty James

**Affiliations:** 1 Department of Psychology, University of Bath, Claverton Down, Bath, Somerset BA2 7AY UK; 2 Bristol Adult CF Centre, Bristol Royal Infirmary, University Hospitals Bristol NHS Foundation Trust, Bristol, BS2 8HW UK

**Keywords:** Cystic fibrosis, Sexual and reproductive health, Sex and relationships education, Pediatric, Adult, United Kingdom

## Abstract

Increasing life expectancy within cystic fibrosis (CF) raises challenges around previously neglected topics such as sexual and reproductive health (SRH). The study aimed to gather retrospective experiences of service provision around SRH to consider the role of the CF service, age of information provision and unmet needs highlighting possible improvements to provision. A mixed-methods retrospective survey-based design was employed. An Adult CF team participated in a consultation session generating survey questions around SRH. A 20-item online survey was constructed and disseminated to adult CF patients. Unmet needs were found in SRH provision in pediatric and adult CF services, with further information required by patients on topics including parenthood and fertility. Results support previous research findings highlighting the need for standardized provision around SRH. Age of SRH provision suggested individual differences in need within the pediatric service. Further research could explore format and specific age of SRH information provision.

## Introduction

Cystic fibrosis is a recessive genetic disorder caused by an abnormality of the CFTR gene, resulting in production of thick mucus particularly in the lungs and pancreas and leading to recurrent infections, breathing difficulties and digestive problems [12]. Prevalence of CF in the UK is around 1 in 2500 births [12], with over 10,000 people currently living with CF in the UK (Cystic Fibrosis Trust [1]).

CF is life-limiting, with median predicted survival in the UK estimated at 45 years (Cystic Fibrosis Trust [1]), although this is increasing yearly, with a median predicted survival of more than 50 years for children born in 2000 [4, 19]). Increased life expectancy raises issues such as pregnancy and parenthood, which were not previously considered possible in CF. As CF is a recessive disorder, both parents must carry the CFTR gene for a child to inherit the disorder, highlighting issues around reproductive decision-making.

### Key SRH Issues in CF

Beyond genetics, several other areas of SRH pose specific issues in CF. A review by Frayman and Sawyer [8] summarized challenges around effectiveness of contraception, STIs, fertility, pregnancy and parenthood and in vitro fertilization (IVF) amongst other areas.

Around 98% of men with CF are infertile (Cystic Fibrosis Trust [2]). Research suggests that male infertility is often discussed at diagnosis but may not be brought up again by teams unless asked [8]. Little conclusive evidence exists for women to suggest that fertility is affected [5, 8]. Despite this, Sawyer et al. [17] found misconceptions about fertility in women with CF adversely influence contraceptive use, highlighting risks of unplanned pregnancy and STDs. Sawyer et al. [16] found that 1 in 3 men at a clinic in Australia believed they did not need to use condoms due to being infertile, highlighting further implications for STDs, although McEwan et al. [13] found no difference in the prevalence of contraceptive use in adults with and without CF.

Research has also highlighted a lack of knowledge and common misconceptions around SRH issues in CF [8, 10]. Gage [9] demonstrated a need amongst female patients for further knowledge in physiological, genetic and psychosocial SRH issues to aid reproductive decision-making. Withers [20] also showed SRH to be a key management issue amongst adolescents with CF, suggesting it should be included in regular check-ups of adolescents’ psychosocial needs. However Sargant et al. [15] found that across several life-limiting conditions, history relating to sexual health was not being recorded for any adolescents in their sample (n = 25), suggesting this does not form part of regular check-ups for adolescents, although findings may be influenced by poor record-keeping.

### SRH Provision

SRH issues specific to CF raise questions about how health services for patients with CF provide this information to patients. Frayman and Sawyer [8] evaluated current SRH provision internationally, highlighting considerable discrepancies in several studies between actual and preferred age by patients of initial discussion about SRH with CF services (with average age of initial discussions ranging from 16 to 17.4 years across studies; compared to preferred age ranging from 13.7 to 14 years). Furthermore, inconsistencies in parental and patient knowledge of SRH in CF were evident, with a high proportion wanting more information, including pediatric patients themselves [14]. One challenge in providing CF-specific SRE is that evidence in some areas such as female fertility and the effects of pregnancy on women’s health with CF is inconclusive and contrasting [8]; yet factors such as these play a role in reproductive decision-making for patients with CF [18] suggesting the importance of accurate and standardized guidance. In line with this, findings of Frayman and Sawyer’s review [8] led to the proposal of a model of service provision to address unmet patient needs and inconsistencies in SRH care, including a timeline of SRE for parents and patients by the CF service. Recent research published since the beginning of the present study supported findings of the review and model with qualitative data from female CF patients and program directors in the US [11]. Themes included the importance of discussing SRH, with care providers initiating conversations, and barriers for patients and providers in discussing SRH, along with preferences for both written information and conversation. A limitation with the sample of program directors was the small size (n = 16) along with demographics; the majority were male with a mean age of 55 years, which may not be representative of clinicians in the CF service who would typically provide SRH information. In addition, no distinction was made between information provided in adult and pediatric services.

### Context of Local Service Provision

Previous projects within the team and nationally, led to the creation of a website for patients with CF around pregnancy and parenthood, however an unmet need had been identified within the pediatric service. Although pediatric clinicians routinely informed male patients about the biological effect of CF on fertility, a standardized procedure for routine SRE about issues such as contraception and sexually transmitted diseases (STDs) was not in place, despite these issues being stipulated in national legislation in schools (Department of Education [3]). The gap in service provision highlighted a need to evaluate the age at which SRE might be provided within the pediatric service, and current patient knowledge and service provision around sex and relationships in CF.

### Aims

- To consider the age at which cystic fibrosis (CF)-specific issues within sexual and reproductive health (SRH) and relationships should be discussed and the role of the CF service in doing this
- To consider whether service provision met patients’ needs according to their experience of sex and relationships education (SRE) throughout the service
- To highlight patient’ need and opinion, and enable the adult and pediatric CF services to make improvements to provision through reflecting on SRE and policies

These aims will be addressed through information gathering about SRE provision within a UK clinical population of adults with CF, in order to compare findings to those gathered in US and Australian populations [8, 11] and answer further questions about the role of both adult and pediatric teams in providing SRE to patients with CF. The study therefore uses a service-based approach, whereby findings are directed toward service evaluation and improvement.

### Research Questions

- Did past service provision in the area of SRH and relationships meet patients’ needs and how this could be improved?
- What age do patients think CF-specific SRH and relationship issues should be discussed and what role does the CF service have in this?

### Design

The study used a retrospective survey-based design with adult patients with CF. Initial consultation with Clinical Psychologists working across pediatric and adult Cystic Fibrosis services identified that due to service limitations and concerns around data collection directly with pediatric patients or their parents, the methodology was revised to gather retrospective information from adult patients with CF.

## Method

### Procedure

#### Phase 1: Service Consultation and Scoping

Following initial scoping, phase one involved a continuing professional development (CPD) session with a Pediatric CF service around CF-specific SRH issues. Members of the multi-disciplinary team (MDT) were presented with a seminal review paper and model of SRH service provision [8] in order to consider the application of the model and scope potential survey questions. Clinician participants took part in a feedback session facilitated by the primary researcher. Represented professions included psychiatry, clinical psychology, nursing and physiotherapy.

#### Phase 2: Questionnaire Design

Based on responses from the MDT and the literature, a 28-item survey was constructed around SRH and relationships within CF. Feedback from several adult patients around readability and comprehension was positive. The survey received ethical approval from the University of Bath’s Psychology Ethics Committee. However, revisions to the questionnaire and methodology were required to gain approval from the Research and Development (R&D) team at the hospital base. Revisions included more multiple-choice questions and fewer free-text questions, with participants offered the option of a face-to-face interview if preferred to an online survey due to the sensitive nature of the topic. During this process, feedback on the survey was gathered through a further CPD slot with an Adult CF team. Concerns were around survey items that may not be relevant to all patients, such as urinary incontinence; and possible recall bias of specific information about age, therefore these questions were removed. Feedback from adult inpatients with CF on a respiratory hospital ward suggested that patients were comfortable with an online survey but appreciated the option of a face-to-face interview, and that the topic of SRH was important to discuss.

Once full approval was gained from the Research and Development Department, the revised 22-item survey was piloted with four adult CF inpatients on the respiratory ward, generally receiving positive feedback. Further concerns were raised around recall, and reluctance to give critical feedback to current care providers. In response to this, retrospective questions included the response item ‘don’t know/can’t recall’ to allow for difficulties with recall, and concerns around critical feedback were addressed through the consent form, ensuring participants were aware that all data was anonymous and could be withdrawn before submission or questions could be left blank. No issues were raised with comprehension or readability.

#### Phase 3: Survey

##### Recruitment Process

An e-mail with an advertisement (in poster format providing information about the study), researcher contact details and a link to the online survey was sent to all adult patients in a UK CF service using an existing database with permission from the service. A follow-up e-mail prompt was sent three weeks later. Where e-mail contact was not possible, paper copies of the survey were sent with a brief summary paragraph about the project, information sheet, consent and debriefing forms, however no copies were received back. In order to increase sample size, patients who were well enough to consent were approached to complete the survey when attending clinics at the CF service and whilst inpatients on the respiratory ward.

##### Participants

Participants were adult patients (N = 223) with an open referral to a UK adult CF service. Response rate was 14%, with a final sample of 31 patients. Low response rate may have been due to the taboo subject of sex and relationships, which some patients may have been uncomfortable discussing. In addition, it is possible that some patients may have struggled to recall their experiences of sex and relationships education therefore may not have responded for this reason. Other possible reasons include the time requirement and current health of patients. The final sample consisted of 17 males and 13 females, with a minimum age range of 18–24 and a maximum age range of 45–54. Demographic data was not received for one participant.

##### Measures

A 22-item questionnaire was devised by the research team and refined through the process described above; therefore no data are available around validity and reliability. Survey questions were based on themes in the literature; refined using expert knowledge from clinicians, R&D committees and patients to determine if the survey fitted with the aims and research questions, suggesting that the survey had content and face validity.

##### Methodology

Participants completed the survey on the Bristol Online Survey website by following the disseminated link. Before starting, participants were given an information sheet with researcher contact details and a statement of participation consent. Participants who consented were directed to the first question; if consent was not gained, the survey redirected to the debriefing statement. The survey included free text boxes; multiple-choice questions and grids, and was anticipated to take 10 to 20 min to complete. Seven questions at the end of the survey collected demographic information, including age of diagnosis. An additional question collected e-mail addresses of those keen to take part in a possible follow-up survey, however this was beyond the scope of the current project. As such, an e-mail was sent to interested participants informing them that they may be contacted at a later date by the service in order to carry out follow-up research. At the end of the survey, final consent was required as upon submission, all data were anonymized and unidentifiable, therefore participants would be unable to withdraw their data after this point. A debrief statement was given including the option to opt out of further e-mail prompts.

## Results

Results from multiple-choice questions were analyzed using descriptive statistics. Demographic results are summarized in Table 1.

**Table 1:** Demographic summary of sample

Category^a^	Response						
Gender	Male	Female					Did not answer (%)
	54.8%	41.9%					3.2
Age	18–24	25–34	35–44	45–54			
	41.9%	25.8%	22.6%	6.5%			3.2
Sexuality	Heterosexual	Gay/lesbian	Bisexual	Other			
	83.9%	3.2%	3.2%	6.5%			3.2
Marital status	Single	Married	Cohabiting	Separated/divorced			
	45.2%	29%	19.4%	3.2%			3.2
Children	Yes	No	Did not answer				
	19.4%	77.4%	3.2%				3.2
Of those that said yes^b^: age of children	0–4	5–9	10–14	15–18	19+		
	14.2%	71.4%	0	14.2%	0		
Age of diagnosis^c^	0–11 m	1–2 yrs	3–4 yrs	5–6 yrs	7–8 yrs	20+ yrs	
	51.6%	16.1%	9.7%	6.5%	3.2%	6.5%	6.5

^a^No. of respondents = 30

^b^No. of respondents = 6

^c^No. of respondents = 29

### Pediatric Service Provision

Generally, participants agreed that advice around SRH and relationships should be provided by the CF team (Table 2), and that historically this has not been very useful (Table 3). Although the largest proportion had felt able to ask the pediatric team about CF issues (40%, although 30% could not recall), the majority were unlikely to have done this (65.5%, with only 6.9% who could not recall). Overall, participants would have liked more information on a range of topics from the pediatric service, suggesting an unmet need (Table 4).

**Table 2:** Role of the pediatric CF service in SRE

Role of the pediatric CF service^a^	Agree/strongly agree		Disagree/strongly disagree		Neither/don’t know	
	*n*	%	*n*	%	*n*	%
Provide CF-specific advice	27	87.1	1	3.2	3	9.7
Provide general advice	21	67.7	4	12.9	6	19.4
Provide updates	16	51.6	5	16.1	10	32.3
Not provide SRE without being asked	7	22.6	14	45.2	10	32.3
Play a role in discussing SRH with partners	19	61.3	4	12.9	8	25.8

^a^No. of respondents = 31

**Table 3:** Support around SRH

Source of support^a^	Very useful		Fairly useful		Not very useful		Don’t know/can’t remember	
	*n*	%	*n*	%	*n*	%	*n*	%
School	3	9.7	12	38.7	14	45.2	2	6.5
Parents	3	9.7	16	51.6	10	32.3	2	6.5
Pediatric CF service	2	6.5	9	29	14	45.2	6	19.4

^a^No. of respondents = 31

**Table 4:** Information provision by the pediatric CF service

Topic of information^a^	Got all the information needed		Would have liked more information/support		Didn’t want this information/support		Not sure/can’t remember	
	*n*	%	*n*	%	*n*	%	*n*	%
Contraception	6	20	12	40	7	23.3	5	16.7
IVF	2	6.7	15	50	7	23.3	6	20
Pregnancy	5	16.7	13	43.3	7	23.3	5	16.7
Fertility	4	13.3	16	53.3	5	16.7	5	16.7
Parenthood	3	10	15	50	5	16.7	7	23.3

^a^No. of respondents = 30

### Adult Service Provision

Most commonly, participants had sought information from the adult CF team around fertility and IVF (Table 5). Out of 19 respondents, the majority of requests were fully met (57.9%) although a large proportion were only partially met (42.1%). Generally participants who had not sought information felt they did not need it (71.4%), whereas just over a quarter of participants (28.6%) preferred to find this out this information from other sources. Most commonly, participants rated service around SRH overall as good (38.7%), followed by fair (32.3%).

**Table 5:** Topics of information sought from the adult CF service

Topic^a^	*n*	%
Contraception	6	33.3
IVF	10	55.6
Fertility	12	66.7
Pregnancy	8	44.4
Parenthood	5	27.8

^a^No. of respondents = 18

### Thematic Analysis of Qualitative Responses

Results from free-text questions were analyzed predominantly through deductive thematic analysis, as themes were anticipated based on previous research findings that shaped questions, although some inductive analysis occurred to capture themes not predicted by the literature. Fourteen initial themes were refined to eight through a process of re-reading responses and codes, and returning to the research question and literature. Within each theme, responses referring specifically to adult or pediatric services were identified.

#### Theme 1

##### Insufficient Information and Awareness About SRH in CF

Several participants reported that they “did not receive any information in pediatrics” and suggested that receiving more information “would have been useful” (participant 16).

I didn’t even know there might be issues until I heard about it in a year 10 science lesson (participant 19)

Some responses suggested frustration at a lack of information:

I would like to point out that the ‘hospital’ response above would be more accurate with a box for: non-existent. (participant 16)

Other responses highlighted the negative emotional impact of not receiving sufficient information:

At age 21 I was tested for fertility… No one really ever spoke to me about the results (infertile). I suspected I should just accept it and get on with it… I spent too long thinking the worst. (participant 19)

I would have loved to receive any information about sexual issues in my early teens; it simply wasn’t discussed. Much to my detriment. (participant 7)

…you don’t realize how much it affects you until you start and go through it all, worrying about things you shouldn’t and would be solved if the cf team spoke more about it all (participant 17)

One participant when reflecting on the outcome of tests with the clinic wrote: “It was almost as if the result was an after though… on reflection this was a huge moment in my life and could have been dealt with better” (participant 19)

#### Theme 2

##### Barriers to Discussing SRH

Participants highlighted key barriers such as being “Too embarrashed (sic) to raise any issues” (participant 7) and feeling it was “awkward to bring up” (participant 1) that prevented them from having conversations about SRH with the CF team.

In particular, they highlighted a desire for the CF team to initiate discussions to overcome barriers:

It should be raised by the staff…many folk are likely to be embarrassed to raise it themselves (participant 16)

One participant highlighted the barrier of cultural background:

…coming from a middle class background it wasn’t the done thing. (participant 7)

Another highlighted a potential barrier of trust in the CF team:

…personally I have to trust that person I couldn’t go to a consultant but a cf nurse are always the best (participant 17)

#### Theme 3

##### Individual Differences (Inductive)

Another interesting pattern in the data was the influence of individual differences, highlighting that “every individual patient is different” (participant 15) and that “… Professionals should gauge the individuals needs and respond appropriately.” (participant 19). This included optionality, with patients suggesting “it would be good to OFFER CF related sex/relationships advice so it is there if someone needs it” (participant 3) but “if the patient does not want to discuss it, they shouldn’t have to” (participant 5). This also included considering different formats for information, for example taking “… a leaflet in a clinic room which gave advice.” (participant 18), as “the option of written information may be preferred by some.” (participant 5).

#### Theme 4

##### Roles and Responsibilities of the CF Team

Several participants highlighted the CF team’s role in developing a supportive relationship with patients as well as providing information.

it’s important to have the cf team by your side ever (sic) step and so you both have a understanding of what you want in your life (participant 17)

I feel i have a large knowledge base & should i have any queries i can count on my CF team to be there for me. (participant 8)

Other participants highlighted the responsibility of the CF team to offer “an open and honest platform for discussion around these topics… Doors for conversation should always be left open.” (participant 19), with patients knowing that information “is important and freely available, judgement-free” (participant 15).

Several participants suggested a role in moving beyond medical information and perspectives, offering “Good advice from a personal point of view not a medical one.” (participant 20) and recognizing that “Psychological health is a huge part of cystic fibrosis” (participant 19), for example psychologists helping “to get you in a place mentally in preparation for IVF and becoming a parent.”(participant 8).

#### Theme 5

##### Content

Participants highlighted when the adult CF team had helpfully provided information on topics such as “information about IVF, pregnancy and the risks to lungs during my late 20’s.” (participant 18). Other topics where further information was required included: “a little bit more info on the IVF front.” (participant 25); “…a federal to be tested for fertility” (participant 1); advice about “going on contraception and explain how pregnancy can affect you” (participant 17); “testing partners for cf gene” (participant 12) and “General help with conception if you want to get pregnant.” (participant 20). One participant suggested that “… the subject of sex, different positions and coping with CF was never really brought up.” (participant 18).

Some participants suggested that they would have liked “A general intro to cf related issues…Maybe at annual review a generic question offering support in this area” (participant 14). Others highlighted content that they had been unaware of or where information was assumed to be known:

There are assumptions made as an adult you are aware about sex and suitable positions to assist with lung conditions. However, this is not true (participant 18)

#### Theme 6

##### Age and Time

A number of participants highlighted the need for information provided to be “age appropriate.” (participant 7), suggesting that “…the right care at the right age can make all the difference” (participant 18).

Some participants suggested that information did not apply to them whilst in pediatric services:

I did not come close to being sexually active until I was 19 (participant 15)

issue of sexual health was not an issue at just 16…more important at adult clinic than pediatric. (participant 12)

Others highlighted the challenge of providing standardized information at the same age to all patients, linking back to the theme of individual differences:

…Some become sexually active earlier than others, which makes it difficult to generalize the timing of the delivery of such information. (participant 15)

No one should be left without the answers they need but at the same time no one should be overloaded with information they might struggle to process at a young age (participant 19)

Participants also highlighted changes over time in the discussion of SRH in the CF service, with one participant stating: “I felt like it was not appropriate or indeed back then the hospitals role” (participant 14), whereas others saw the lack of information in the past as a missed opportunity:

I would have loved to receive any information about sexual issues in my early teens; it simply wasn’t discussed (participant 7)

… 25 + years ago this was not even considered… just did not exist whilst growing up through peads [sic] service (participant 14)

#### Theme 7

##### SRH as Part of CF Overall (Inductive)

The position of SRH as an important part of the broader picture of CF was not anticipated from previous research. Participants suggested that “it’s important to know about everything because of your illness” (participant 17), including SRH in order to “have more of a full understanding on my disease” (participant 16).

One participant considered SRH simply as one part of their overall CF, suggesting that “guidance from the hospital are purely how CF deviates me from the norm, and this stretches to most aspects of my adult life.” (participant 15), whereas others placed SRH in a position of high importance within their overall care: “Psychological health is a huge part of cystic fibrosis” (participant 18).

In contrast to the rest of the theme, one participant made a clear distinction between SRH and other aspects of CF:

clinic was to talk about lungs and digestion, not sex (participant 16)

#### Theme 8

##### The use of Other Sources of Information

A number of participants highlighted that rather than the CF team being responsible for SRH, sources such as “parents should be responsible for educating their children on sex and reproduction” (participant 2), as “General sex/relationships advice is supplied by GPs, school sessions, school nurses, adverts, online etc. so I don’t think it’s necessary on top of this to have it in CF Clinic!” (participant 3).

Other sources of information included school:

I heard about it in a year 10 science lesson (participant 19)

Media, for example TV programs such as “Embarrassing bodies” (participant 17) or “online info” (participant 29) from “…the internet…when it became more prevalent at home.” (participant 16)

Other medical professionals, such as “Doctors” (participant 21) and “Gynecology” (participant 17).

One participant highlighted the informal distribution of information around SRH via peers:

It was mostly friends, rumor and suggestion. (participant 16)

## Discussion

Results generally supported previous findings around insufficient and variable service provision outlined in Frayman and Sawyer’s review [8]. Patients reported a lack of information provision in pediatric and adult services, which in some cases had resulted in negative effects such as spending “too long thinking the worst” about being unable to have children due to infertility (participant 19). This highlights psychological consequences of insufficient service provision around SRH, in addition to other risks such as reduced contraceptive use [6, 16], thus supporting the importance of a standardized approach.

In response to the first research question, findings suggest that pediatric provision generally did not meet service user need. Participants most frequently described it as ‘not very useful’, reporting that they wanted more information on a range of SRH topics. This supports evidence directly from pediatric patients [14], as well as parent-reported knowledge from pediatric services [7] suggesting a reliable finding despite a retrospective approach to data collection. Barriers to information seeking such as embarrassment and a desire for the clinicians working within the CF service to initiate conversations highlight the role of the CF service in meeting patient need in this area. Patients did not feel that information provision should be dependent on a patient request, but should be provided as standard within the service, including refreshers of information over time, in line with Frayman and Sawyer’s model [8] of service provision.

In contrast to previous research, some respondents did not feel that SRE was relevant for them whilst in the pediatric service, with reasons including the age at which they became sexually active. This highlights the importance of sensitivity to individual differences when providing SRE. In line with this, previous research has advocated an individualized approach in terms of age and format [11, 20], however suggests that giving patients the option of SRH information is still necessary.

Findings indicating that patient requests for SRH information were fulfilled less than two-thirds of the time highlight a shortfalling in service provision even when patients overcame identified barriers to initiate the conversation. Some participants reported not seeking information from the adult service as they did not require the information on certain topics. Based on the demographic spread, where participants most commonly fell in the 18–24 age range and did not have children, it could be hypothesized that the proportion of patients seeking information in the future may increase as topics become applicable to them, for example the two most commonly requested topics of fertility and IVF. In addition, findings in theme 7 that patients tended to view SRH as an important part of their overall understanding of CF emphasize the importance of prioritizing service provision in this area. Furthermore, one-fifth of participants reported seeking information from other sources rather than the CF team. Barriers identified by participants to seeking information may lead them to use other sources, therefore reducing these barriers, as well as increasing availability of information, may increase the level of information sought and the prevalence of discourse around SRH in services.

### Service Implications

Implications for health service practice suggest a need for improvement in consistency and overall provision of information about SRH, particularly in a pediatric setting. Patients also reported that refreshers of information would be beneficial, suggesting that information provision should be ongoing to fit with patient need, rather than only provided on one occasion and not repeated. In addition, findings highlighted an important role for the CF service, particularly the pediatric team, in developing a strong relationship with patients and instigating conversations about SRH issues in order to overcome barriers facing patients in discussing these issues, such as embarrassment and awkwardness.

### Limitations

Reliance in this study on self-reported retrospective information from adult patients may reduce reliability due to difficulties with recall and memory bias. The sensitivity of the research topic may have created a bias toward participants who felt more able to discuss SRH or those with strong views on the topic, as well as a possible bias in answers given in line with concerns about criticizing a current care provider. As such, results may not be representative of all patients with CF from within the identified service or wider afield.

## Conclusions and Further Research

This study contributes qualitative information about service provision within pediatric and adult CF services in the UK. Despite reliance on retrospective data, the theme of ‘change over time’ indicated that retrospective reflection enabled some participants to consider information that would have been useful with hindsight, that they may not have realized they needed at the time. This is therefore a useful finding as a result of retrospective methodology. Further research could explore the information needs of both pediatric patients and their parents, enabling data to be collected on the specific age of provision. In addition, research around overcoming barriers to information seeking by patients and provision by clinicians would build on the work conducted by Kazmerski et al. [11]. Finally, further research could explore the format and frequency of SRE provision, building on findings around refreshers and the usefulness of written information. These questions could be answered through consultation with patients, perhaps in the form of interviews or electronic focus groups, for example through video calls, to overcome the risk of cross-infection when patients meet in person.
